# Structural Characterization of Polygonatum Cyrtonema Polysaccharide and Its Immunomodulatory Effects on Macrophages

**DOI:** 10.3390/molecules29092076

**Published:** 2024-04-30

**Authors:** Ruiding Wen, Lu Luo, Runcheng Zhang, Xudong Zhou, Wei Wang, Limin Gong

**Affiliations:** TCM and Ethnomedicine Innovation & Development International Laboratory, School of Pharmacy, Hunan University of Chinese Medicine, Changsha 410208, China; wruiding@163.com (R.W.); lulu1209jinx@163.com (L.L.); runchengzhang163@163.com (R.Z.); xudongzhou999@163.com (X.Z.)

**Keywords:** *Polygonatum cyrtonema*, polysaccharide, structure characterization, immunomodulatory activity

## Abstract

A neutral *Polygonatum cyrtonema* polysaccharide (NPCP) was isolated and purified from *Polygonatum cyrtonema* by various chromatographic techniques, including DEAE-52 and Sephadex-G100 chromatography. The structure of NPCP was characterized by HPLC, HPGPC, GC-MS, FT-IR, NMR, and SEM. Results showed that NPCP is composed of glucose (55.4%) and galactose (44.6%) with a molecular weight of 3.2 kDa, and the sugar chain of NPCP was →1)-α-D-Glc-(4→1)-β-D-Gal-(3→. In vitro bioactivity experiments demonstrated that NPCP significantly enhanced macrophages proliferation and phagocytosis while inhibiting the M1 polarization induced by LPS as well as the M2 polarization induced by IL-4 and IL-13 in macrophages. Additionally, NPCP suppressed the secretion of IL-6 and TNF-α in both M1 and M2 cells but promoted the secretion of IL-10. These results suggest that NPCP could serve as an immunomodulatory agent with potential applications in anti-inflammatory therapy.

## 1. Introduction

Macrophages are innate immune cells widely distributed in blood and tissues possessing diverse functions such as phagocytosis, killing pathogens, activating other immune cells, and promoting tissue damage repair. Macrophages are highly plastic and can normally be activated into two different polarizing phenotype: M1 macrophages induced by LPS or INF-γ (classically activated macrophages) and M2 macrophages induced by IL-4 or IL-13 (alternatively activated macrophages) [1]. M1 macrophages express biomolecules CD80 and CD86 and secrete pro-inflammatory cytokines such as NO, TNF-α, IL-6, and IL-1β, presenting immune-stimulating activities. M2 macrophages express CD206 and CD163 molecules and secrete immunosuppressive cytokines such as IL-10 and TGF-β to downregulate the immune response, inhibit inflammation, and promote tissue repair and angiogenesis. The dynamic balance between M1 and M2 macrophages is crucial for maintaining internal homeostasis and the treatment of diseases. In many inflammatory diseases, such as rheumatoid arthritis (RA) [2,3] and inflammatory bowel disease [4], the balance of M1/M2 macrophages is disrupted, and the increase in the ratio of M1 macrophages leads to aggravation of the inflammatory response and the development of the disease. Tumor-associated macrophages (TAMs) in the tumor microenvironment tend to be M2 polarization [5,6] and secrete cytokines such as IL-10 and TGF-β to suppress immune cells and stimulate angiogenesis [7] and chemoresistance [8] to promote tumor invasion and metastasis, which usually results in a poor prognosis for the patient. Zhang et al. isolated a polysaccharide from *Plantago asiatica* and demonstrated that it did not directly inhibit 4T1 cells but indirectly suppressed their migration by inhibiting M2 polarization and promoting M1 polarization of macrophages [9]. *Pseudostellaria heterophylla* polysaccharides have also been reported to activate NF-κB and MAPK signaling pathways to convert M2 macrophages into M1 macrophages, thereby inhibiting tumor growth [10]. In addition, Yuan et al. injected CpG-DNA combined with anti-IL-10r Ab into mouse Lewis tumors, successfully promoted TAM phenotype reversal from M2 to M1, and significantly inhibited tumor cell metastasis [11]. These findings suggested that adjusting the polarization of macrophages may be an effective strategy in the treatment of inflammatory diseases and tumors.

Plant polysaccharides, a natural macromolecule, were reported to have diverse biological activities, such as anti-oxidation [12], anti-inflammatory [13], anti-tumor [14], hypoglycemic [15] and immune regulation [16]. Especially in immune regulation, many polysaccharides have been proven to be immune enhancers, such as polysaccharides isolated from *Lonicera japonica* can enhance the activity of macrophages and NK cells in cyclophosphamide-induced immunosuppressive mice [17] and polysaccharide from *Helicteres angustifolia L*. can promote the proliferation of spleen cells in 4T1 tumor model mice and increase the percentage of CD4^+^T and CD8^+^T cells, thus exerting antitumor effects [18].

The genus *Polygonatum*, which belonged to the family Asparagaceae, is widely distributed in the northern hemisphere [19]. As a combination of food and medicine, it has a long history of application in traditional usages. With the development of the medical field, the genus *Polygonatum* has received increasing attention for its health-promoting effect. Polysaccharides, as a main constituent of *Polygonatum*, have also been extensively studied. So far, numerous polysaccharides have been isolated and identified from *Polygonatum*, exhibiting diverse activities, including possessing anti-oxidant [20], anti-inflammatory [21], anti-fatigue [22], anti-cancer [23], and immunomodulatory [24] effects. The current research on *Polygonatum* polysaccharides mainly focuses on *Polygonatum sibiricum*, *Polygonatum kingianum*, and *Polygonatum cyrtonema*. Polysaccharides extracted from *Polygonatum sibiricum* have been shown to inhibit LPS-induced M1 polarization of macrophages and promote their polarization to M2, thus exerting anti-inflammatory effects [25]. However, the effects of *Polygonatum* polysaccharides on M2 macrophages are rarely reported. In the present study, a neutral *Polygonatum cyrtonema* polysaccharide (NPCP) was purified from *Polygonatum cyrtonema,* and the structure of NPCP was characterized by molecular weight determination, monosaccharide composition analysis, FT-IR spectra, and NMR spectra. In addition, we investigated the effect of NPCP on LPS-induced M1 polarization of macrophages and IL-4- and IL-13-induced M2 polarization in vitro.

## 2. Results

### 2.1. Purification and Identification of NPCP

**Figure 1:**
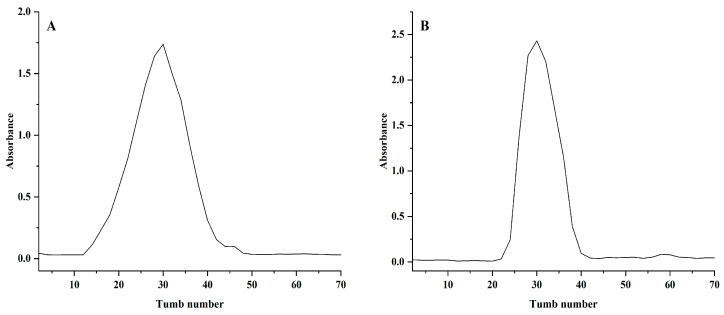
DEAE-52 cellulose column elution curves for NPCP (**A**) and Sephadex G-100 column elution curve for NPCP (**B**).

As shown in Figure 1, the crude polysaccharide was further separated and purified by a DEAE-52 cellulose column, and Sephadex G-100 column, and the eluted fraction in deionized water was collected. After concentration, dialysis, and lyophilization, the neutral *Polygonum cyrtonema* polysaccharide NPCP was obtained.

**Figure 2:**
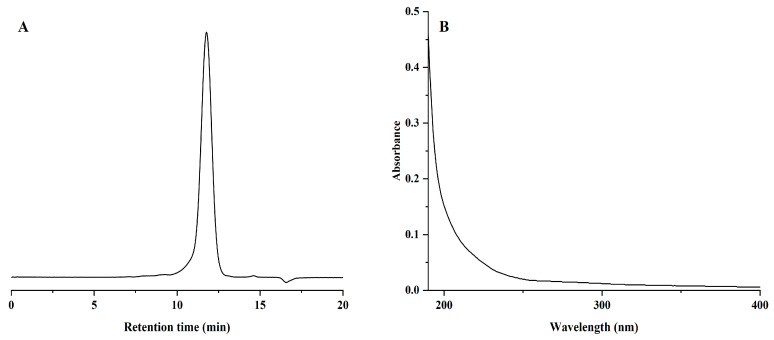
Chromatogram of NPCP molecular weight analysis (**A**) and UV spectra of NPCP (**B**).

**Figure 3:**
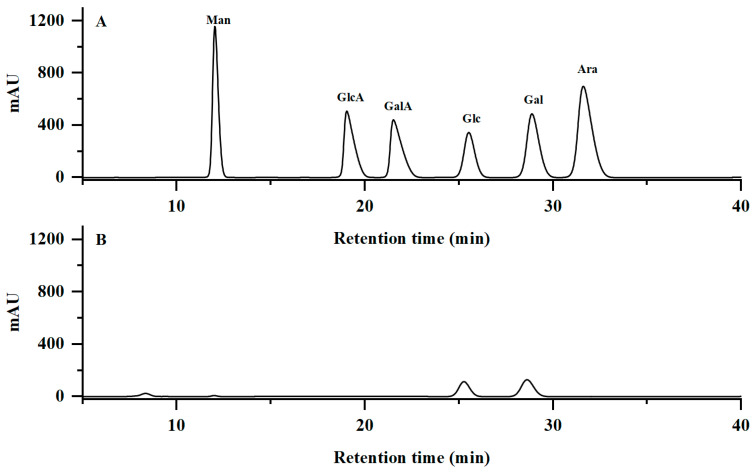
Chromatograms of mannose (Man), glucuronic acid (GlcA), galacturonic acid (GalA), glucose (Glc), galactose (Man), arabinose (Ara) standards (**A**) and NPCP (**B**).

As shown in Figure 2A, NPCP presented a symmetrical single peak, indicating that NPCP was a homogeneous polysaccharide. According to the retention time and calibration curve of the dextran standard (Figure S1), the molecular weight of NPCP was calculated to be 3.2 kDa. The UV spectra of NPCP are shown in Figure 2B. No significant absorption at 260 nm and 280 nm, indicating that no protein and nucleic acid remained in NPCP. Figure 3 shows the monosaccharide composition analysis of NPCP. The results showed NPCP was composed of glucose (55.4%) and galactose (44.6%).

### 2.2. Methylation Analysis

The glycosidic linkage mode of NPCP was determined by methylation and GC-MS analysis. After methylation, hydrolysis, reduction, and acetylation, four derivatives of NPCP were obtained (Figures S2 and S3). As shown in Table 1, the four derivatives are 2,3,4,6-Me_4_-Galp, 2,4,6-Me_3_-Galp, 2,3,6-Me_3_-Glcp, 2,3,4,6-Me_4_-Glcp, respectively. These methylation products indicate that the structure of NPCP contains T-linked Galp, 1,3-linked Galp, 1,4-linked Glcp, and T-linked Glcp.

### 2.3. Scanning Electron Microscopy Analysis of NPCP

**Figure 4:**
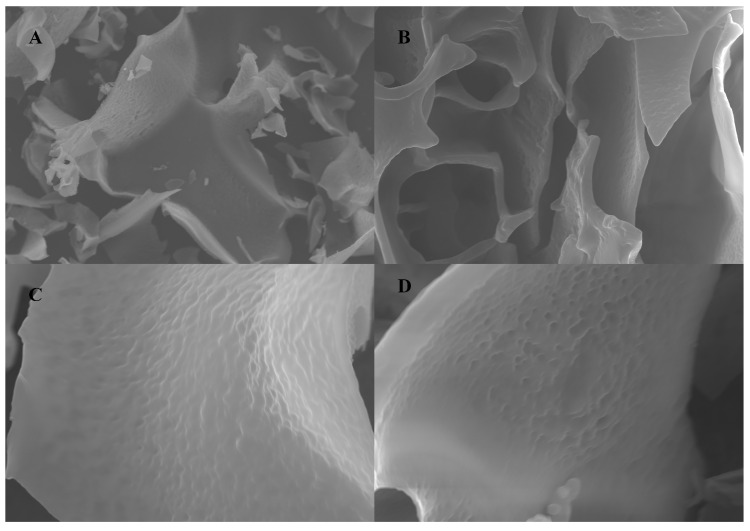
Scanning electron microscopy images of NPCP (**A**) ×500, (**B**) ×1000, (**C**) ×2000, (**D**) ×3000.

The SEM images of NPCP are shown in Figure 4. At 500× and 1000× magnifications, NPCP exhibits a state of fragmented accumulation with a smooth surface. Many tiny pits on the surface of NPCP can be observed at 2000× and 3000× magnifications. These properties may be due to the cavitation effect of sonication during polysaccharide extraction [26,27].

### 2.4. FT-IR Analysis

**Figure 5:**
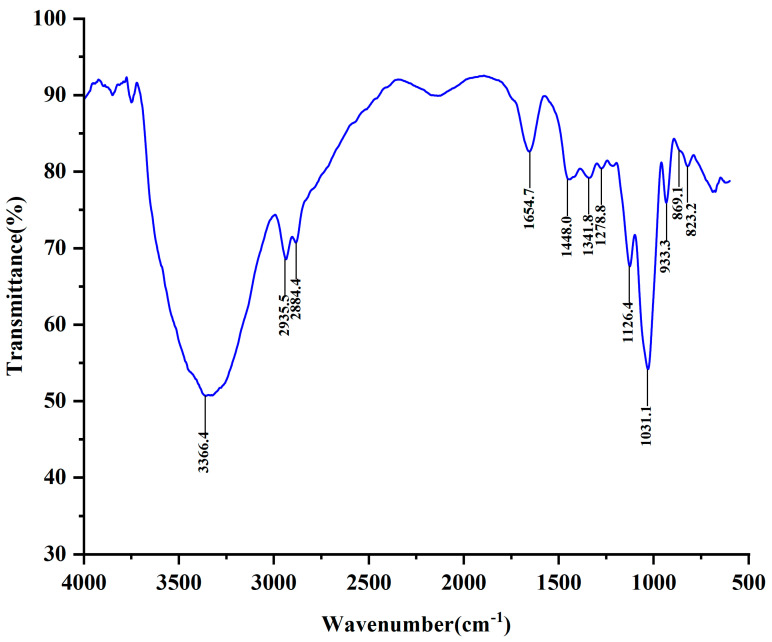
Attenuated total reflection Fourier-transform infrared spectrum of NPCP in the wavelength range of 4000–600 cm^−1^.

The infrared spectrum of NPCP is illustrated in Figure 5. The strong and broad absorption peak at 3366.4 cm^−1^ represents the stretching vibration of O-H in the polysaccharide sugar ring, and the peaks at 2935.5 cm^−1^ and 2884.4 cm^−1^ represent the stretching vibration of C-H [24]. No obvious absorption at 1730 cm^−1^ indicated that there was no uronic acid in NPCP. The absorption peak at 1654.7 cm^−1^ was classified as the stretching vibration of C=O, and the absorption peak near 1488.0–1248.8 cm^−1^ was assigned to the stretching vibration of C-H in -CH_2_ [28]. The stretching vibration of C-H in -CH_2_ and the absorption peaks at 1026.4 cm^−1^ and 1031.1 cm^−1^ were the stretching vibrations of the C-O-C glycosidic bond, the absorption at 933.3 cm^−1^ represented the β-glycosidic bond [29], and the absorption at 823.2 cm^−1^ represented the α-glycosidic bond [30], indicating that both the α and β sugar configurations existed in NPCP.

### 2.5. NMR Analysis

**Figure 6:**
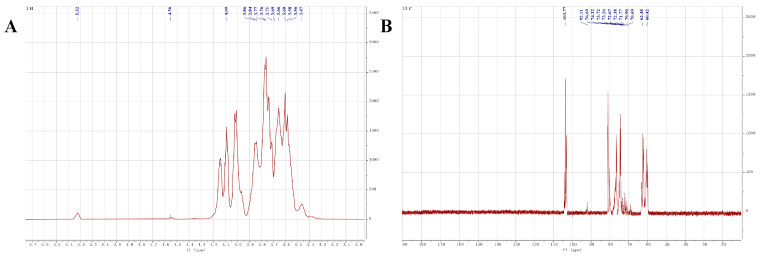
The ^1^H NMR (600 MHz) spectra (**A**) and ^13^C NMR (150 MHz) spectra (**B**) of NPCP in D_2_O.

**Figure 7:**
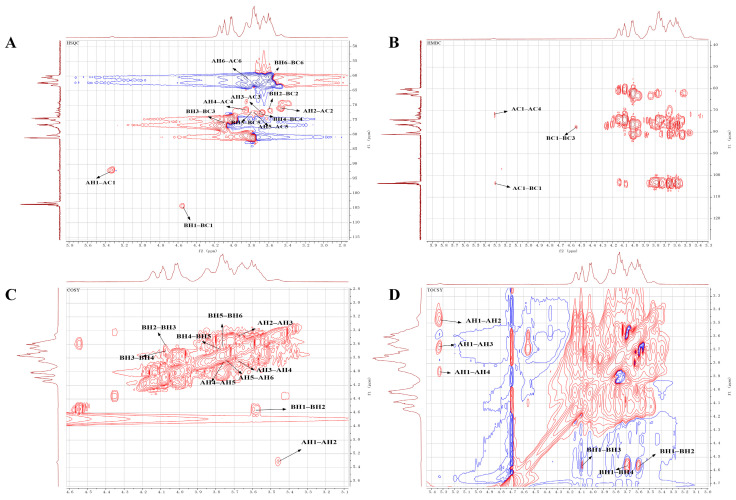
The chemical structure of NPCP elucidated by 2D NMR. The HSQC (**A**), HMBC (**B**), ^1^H-^1^H COSY (**C**), and TOCSY (**D**) spectra of NPCP in D_2_O.

The structural characteristics of NPCP were further analyzed by using 1D and 2D NMR spectra. All spectra are shown in Figure 6 and Figure 7, and the corresponding proton and carbon chemical shifts are listed in Table 2. The characteristic signal of uronic acid was not detected at 170 ppm in the ^13^C NMR spectrum, indicating that NPCP is a neutral polysaccharide. Two anomeric proton and carbon signals, 5.32/92.11 ppm (residue A) and 4.55/103.77 ppm (residue B), were observed in the HSQC spectra (Figure 7A). The anomeric proton signals in 4.2–4.8 ppm are generally attributed to β-configuration, and the signals in 5.1–5.8 ppm are attributed to α-type [31]. Combined with the results of monosaccharide composition and methylation analysis and previous reports [32,33], residue A was assigned as →4)-α-D-Glcp-(1→. The chemical shifts of H2-H6b can be determined as 3.47, 3.66, 3.86, 3.76, 3.84, and 3.69 ppm according to COSY (Figure 7C) and TOCSY (Figure 7D) spectra, respectively. The corresponding chemical shifts of C2, C3, C4, C5, and C6 were 70.69, 72.67, 73.72, 73.26, and 60.42 ppm according to the HSQC spectrum. Residue B with an anomer proton and carbon signal of 4.56/103.77 ppm was assigned to →3)-β-D-Galp-(1→. The peak crossing signals of 4.56, 3.61, 4.07, 3.69, 3.77, 3.66, and 3.58 ppm can be observed in the COSY and TOCSY spectra, which are the signals of H1-H6b. The corresponding C2−C6 chemical shifts of residue B can be observed from HSQC spectra as 71.96, 76.69, 72.81, 72.51, and 62.48 ppm.

**Figure 8:**
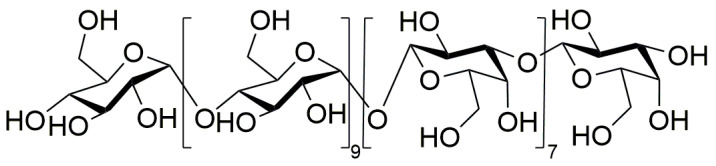
The deduced chemical structure of NPCP.

In the HMBC spectrum (Figure 7B), three cross-peaks (5.31/71.73, 4.55/76.60, and 5.31/103.82 ppm) were observed. These signals belonged to H1 (5.31), C4 (71.73) of residue A and H1 (4.55), C1 (103.82), and C3 (76.60) of residue B, respectively. These results indicate the presence of fragments →4)-α-D-Glcp-(1→4)-α-D-Glcp-(1→ and →3)-β-D-Galp-(1→3)-β-D-Galp-(1→ and →4)-α-D-Glcp-(1→1)-β-D-Galp-(3→ in NPCP. Based on the above data, combined with the molecular weight determination of NPCP and the monosaccharide composition analysis results, the structure of NPCP can be inferred as α-D-Glcp-(1→(4)-α-D-Glcp-(1)_9_→(1)-β-D-Galp-(3)_7_→1)-β-D-Galp (Figure 8).

### 2.6. NPCP Enhanced the Proliferation and Phagocytosis of RAW264.7 Cells

**Figure 9:**
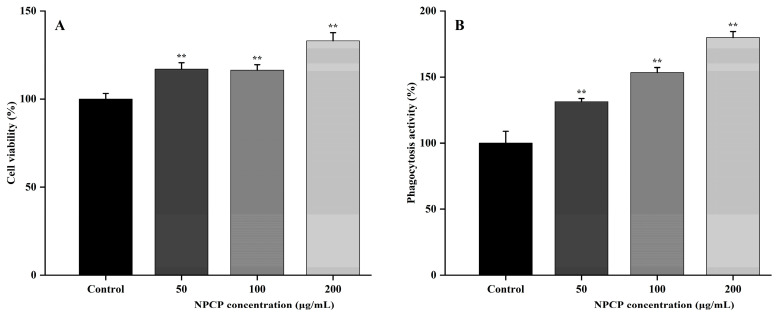
The effect of NPCP on the proliferation of macrophages detected by CCK8 assay (**A**) and the phagocytic activity of macrophages detected by neutral red assay (**B**). Values were presented as mean ± SD (*n* = 3) (** *p* < 0.01, compared to control group).

The effects of NPCP on the viability and phagocytosis of RAW264.7 cells were detected by the CCK8 assay and the neutral red phagocytosis assay. As shown in Figure 9A, the viability of RAW264.7 cells was significantly increased after NPCP (50–200 μg/mL) treatment for 48 h, and 200 μg/mL NPCP had the strongest effect.

Figure 9B showed that the phagocytic activity of RAW264.7 cells was significantly enhanced in a dose-dependent manner after treatment with NPCP (50–200 μg/mL) for 24 h. The results showed NPCP had no inhibitory effect on the proliferation of macrophages and significantly increased the phagocytic capacity of macrophages.

### 2.7. Effects of NPCP on M1 Macrophages

**Figure 10:**
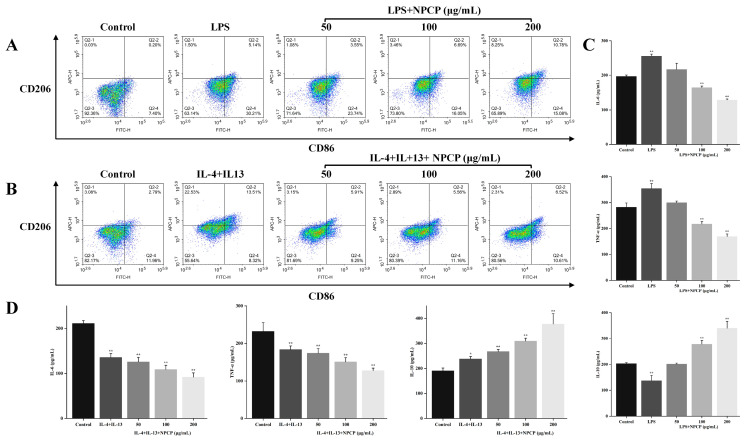
Effect of NPCP on the expression of CD86 and CD206 in M1 (**A**) and M2 (**B**) macrophages. Secretions of IL-6, TNF-α, and IL-10 in M1 (**C**) and M2 (**D**) macrophages after treatment with NPCP for 24 h. Values were presented as mean ± SD (*n* = 3) (* *p* < 0.05; ** *p* < 0.01, compared to control group).

In order to investigate the impact of NPCP on M1 macrophages, macrophages were stimulated with LPS for 24 h and then treated with NPCP for another 24 h. The expression levels of CD86 and CD206 in each group were assessed using flow cytometry. As shown in Figure 10A, the expression of M1 macrophages marker CD86 was obviously increased after LPS treatment (30.21%) compared with the control group (7.40%). At the same time, the levels of proinflammatory cytokines IL-6 and TNF-α secreted by M1 macrophages were increased (Figure 10C), indicating that LPS successfully induced the polarization of macrophages to M1 macrophages. After 24 h of NPCP treatment, the proportion of CD86 in macrophages was significantly reduced, and the effects of 100 mg and 200 mg NPCP on CD86 expression were comparable (Figure 10A), indicating that NPCP has the ability to inhibit LPS-induced macrophages M1 polarization. Moreover, in comparison to the LPS group, 200 μg/mL NPCP also enhanced M2 macrophages marker CD206 expression in macrophages. These results suggest that NPCP could inhibit M1 polarization and partially promote M2 polarization in LPS-treated macrophages (Figure 10A).

Compared to the control group, LPS treatment resulted in an upregulation of IL-6 and TNF-α and a downregulation of IL-10 in macrophages (Figure 10C). After adding NPCP treatment, the levels of IL-6 and TNF-α decreased, and the level of IL-10 increased in a dose-dependent manner (Figure 10C). These findings demonstrated that NPCP could reverse the increase in pro-inflammatory factors and the decrease in anti-inflammatory factors in macrophages after LPS treatment, suggesting that NPCP has application value in the field of anti-inflammation.

### 2.8. Effects of NPCP on M2 Macrophages

Figure 10B showed that the expression of CD206 in macrophages treated with IL-4 and IL-13 for 24 h was significantly increased (22.08%) compared with the control group (3.08%), indicating that IL-4 and IL-13 successfully induced the polarization of macrophages to M2 type. After treatment with NPCP at concentrations of 50–200 mg/mL, the expression of CD206 in macrophages decreased to 3.15%, 2.89%, and 2.31%, respectively, indicating that NPCP could inhibit the M2 polarization of macrophages induced by IL-4 and IL-13. Meanwhile, after NPCP treatment there was no significant increase in the expression of CD86 compared with the IL-4 and IL-13 treatment group, indicating that the number of M1 macrophages did not increase after NPCP treatment. Furthermore, the secretion level of the anti-inflammatory cytokine IL-10 secreted by M2 macrophages was significantly upregulated, while the secretion level of pro-inflammatory cytokines IL-6 and TNF-α was significantly downregulated in IL-4 and IL-13-treated macrophages. In addition, NPCP (50–200 μg/mL) treatment for 24 h further reduced the levels of IL-6 and TNF-α and increased the level of IL-10 in a dose-dependent manner (Figure 10D).

The expression of CD206 in NPCP-treated M2 macrophages returned significantly to the normal level, indicating that NCPC exerted a potent inhibitory effect on IL-4 and IL-13-treated macrophages M2 polarization. However, the level of anti-inflammatory cytokine IL-10 secreted by M2 macrophages was not decreased but increased in response to the inhibitory effect of NPCP, which indicated that NPCP promoted IL-10 secretion while inhibiting M2 polarization of macrophages. Additionally, there was no significant change in the expression of CD86, and the reduction in IL-6 and TNF-α suggested that NPCP did not promote M1 polarization in macrophages.

## 3. Discussion

In this study, we isolated and identified a neutral polysaccharide NPCP from *Polygonatum cyrtonema*. NPCP is a low-molecular-weight polysaccharide characterized by an α-1,4-linked Glcp and β-1,3-linked Galp skeleton and exhibiting distinct structural compared to other *Polygonatum* polysaccharides. In addition, the immunomodulatory effects of NPCP on macrophages were investigated in vitro using CCK8 assay, neutral red assay, and flow cytometry. Macrophages play an essential role in innate immunity, ensuring homeostasis by phagocytizing substances such as dead cells and pathogens. The phagocytosis ability of macrophages was significantly enhanced after NPCP treatment, and the CCK8 experiment results showed that NPCP could promote the proliferation of macrophages at dosages of 50–200 mg/mL, indicating the pharmacologically therapeutic safety of NPCP.

Macrophages can exhibit pro-inflammatory phenotype (M1) and anti-inflammatory phenotype (M2) under different conditions and show dynamic changes with the development of the disease. In the present study, we investigated the effects of NPCP on M1 macrophages induced by LPS and M2 macrophages induced by IL-4 and IL-13. We examined the expression of CD206 and CD86 and the secretion of IL-6, TNF-α, and IL-10 in NPCP-treated M1 and M2 macrophages. Our results showed that NPCP inhibited the expression of CD86 and the secretion of IL-6 and TNF-α in M1 macrophages, indicating NPCP could inhibit macrophages M1 polarization induced by LPS. IL-6 and TNF-α are typical cytokines secreted by M1 macrophages; in the early stage of infection and tissue injury, IL-6 and TNF-α were rapidly produced to stimulate the immune response. However, the excessive production and accumulation of IL-6 and TNF-α lead to persistent inflammatory responses that cause damage to the body. The serum levels of IL-6 and TNF-α are elevated in RA patients, and this elevation is related to the degree of inflammation in the disease [34,35]. TNF-α has been demonstrated to promote proliferation and inhibit the apoptosis of synovial cells, resulting in pannus formation and the destruction of joints in patients with RA [36]. In addition, TNF-α and IL-6 can also promote the formation of foam cells, which increases the risk of atherosclerosis in RA patients [37]. Plant polysaccharides from *Saposhnikovia divaricate* and *Dendrobium huoshanense* have been shown to possess therapeutic effects on RA by reducing the production of inflammatory factors and inhibiting the proliferation of fibroblast-like synoviocyte (FLS) cells. NPCP inhibited LPS-induced macrophages M1 polarization and the secretion of proinflammatory cytokines IL-6 and TNF-α while promoting IL-10 secretion and macrophages M2 polarization. These results suggest that NPCP may have potential in the development of functional foods or drugs for inflammatory disease treatment such as RA in the future.

IL-10 is a pleiotropic cytokine produced by various cells, which has the effects of inhibiting inflammatory response, promoting tissue injury repair [38], and immunosuppression [39]. Our results showed that NPCP inhibited CD206 expression in M2 macrophages but had no significant effect on the expression of CD86, which indicated NPCP only inhibited the M2 polarization of macrophages and did not promote the M1 polarization. Additionally, NPCP dose dependently decreased IL-6 and TNF-α levels and promoted IL-10 secretion. This result may be attributed to the heterogeneity of M2 macrophages, which can further be classified into four subtypes: M2a, M2b, M2c, and M2d [40,41]. NPCP treatment suppressed M2a macrophages, resulting in a downregulation of CD206. Meanwhile, it enhanced the function of M2c macrophages, leading to the upregulation of IL-10 levels. Compared with M2a cells, M2c cells exhibited superior ability in reducing lung and kidney injury and possessed a better effect in reducing fibrosis [42,43]. These findings suggest that NPCP may serve as a potential targeted regulator of macrophages M2a polarization, and the related mechanisms need further research and clarification.

## 4. Materials and Methods

### 4.1. Materials and Reagents

*Polygonatum Cyrtonema* were obtained from Xupu County, Hunan Province, China. DEAE-52 cellulose, Sephadex G-100, and different monosaccharide standards (mannose, glucose, galactose, glucuronic acid, and galacturonic acid) were purchased from Yuanye Bio-Technology (Shanghai, China). Different molecular weight dextran was purchased from Aladdin Biochemical Technology Co., Ltd. (Shanghai, China). Acetonitrile was purchased from Sigma-Aldrich (St. Louis, MO, USA). D_2_O (D, 99.9%) was purchased from Chemiejoy (Shanghai, China). All other reagents were analytically pure and purchased from Sinopharm Chemical Reagents Co., Ltd. (Shanghai, China).

### 4.2. Extraction, Isolation, and Purification of Polysaccharide from Polygonatum cyrtonema

The dried and crushed *Polygonatum cyrtonema* was refluxed twice with petroleum ether (1:10, *w*/*v*) to remove the lipid-soluble components and then extracted twice with deionized water at 70 °C under ultrasonic conditions. The extract was then collected and the precipitate was concentrated with a fourfold volume of absolute ethanol. The precipitate was dissolved in deionized water and mixed with Sevag reagent (n-butanol: chloroform = 1:4, *v*/*v*) to remove proteins. Then, the aqueous solution was dialyzed and freeze-dried to obtain the crude polysaccharides.

The crude polysaccharides (1.0 g) were dissolved in deionized water (20 mg/mL), submitted to a DEAE-52 cellulose column (4.6 × 45 cm), and eluted with deionized water. The eluate was collected at 10 mL per tube and the polysaccharide contents were determined by the anthrone sulfuric acid method. The polysaccharide-containing fraction was concentrated, dialyzed, lyophilized, and then submitted on a Sephadex G-100 column (2 × 120 cm) at 30 mg/mL and eluted with deionized water. The eluate was collected at 5 mL per tube and detected by the anthrone sulfuric acid method. All tubes containing polysaccharides were concentrated, dialyzed, and freeze-dried to obtain refined polysaccharides.

### 4.3. Molecular Weight Analysis

The molecular weight was determined by high-performance gel permeation chromatography with an Alliance E2695 system (Waters, Milford, MA, USA) equipped with a TSK gel GMPW_XL_ column (7.8 mm × 300 mm, Tosoh, Japan) and a RID (Waters, USA) detector. Samples (4 mg/mL) were injected into the system in a volume of 50 μL; the column temperature was set at 35 °C, and pure water was used as the mobile phase to elute at a flow rate of 0.8 mL/min. The molecular weight of samples was evaluated with a calibration curve of Dextran T-series standard.

### 4.4. Monosaccharide Composition Analysis

A total of 5 mg NPCP was hydrolyzed with 4 mL 2M TFA for 3 h at 120 °C. Excess TFA was removed by rotary evaporation, and the hydrolysate was derived with PMP (1-phenyl-3-methyl-5-pyrazolone). Derivatives were analyzed on a Shimadzu HPLC system equipped with an SPD-M40 detector and a Shim-pack GIST C18 column (4.6 × 150 mm) (Shimadzu, Kyoto, Japan), eluted with 83% PBS (0.05 M, pH 6.5) and 17% acetonitrile (*v*/*v*) at a flow rate of 1.0 mL/min, and monitored by UV absorbance at 254 nm.

### 4.5. Methylation Analysis

Methylation is a crucial technique for investigating the linkage mode of glycosidic bonds in polysaccharides. After all the free hydroxyl groups in polysaccharides are methylated and then hydrolyzed by acid and acetylated, derivatives of glycolipids are obtained. Finally, the ion fragments of these derivatives are analyzed using GC-MS to infer the linkage mode of glycosidic bonds based on the ionization behavior of glycolipids.

NPCP was dissolved in 2 mL DMSO and then reacted with 10 mg NaOH for 30 min. After that, 2 mL iodomethane was added, and the reaction was allowed to run for 12 h in the dark. The reaction was subsequently terminated with deionized water, and the product was dialyzed for 48 h in deionized water. Methylation products were extracted with chloroform and hydrolyzed with 4 mL 2 M TFA for 3 h at 120 °C. Next, 1 mL (20 mg/mL) NaBH_4_ was added to the hydrolysate and incubated for 4 h at room temperature. The reaction was then terminated with glacial acetic acid and evaporated to dryness, 1 mL acetic anhydride and 1 mL pyridine were added to the reaction product, and the reaction was carried out for 2 h at 100 °C. Then, 1 mL acetic anhydride and 1 mL pyridine were added and reacted at 100 °C for 2 h. The acetylation products were extracted with chloroform and analyzed by GC-MS (Shimadzu QP2010, Japan) equipped with DB-5MS column (60 m × 0.25 mm × 0.25 μm). The initial column temperature was 60 °C and maintained for 5 min, then increased to 240 °C at 4 °C/min and maintained for 10 min. The ion source temperature was 250 °C, with helium as the carrier gas.

### 4.6. Scanning Electron Microscopy (SEM) Analysis

A small amount of polysaccharide was adhered with conductive film and placed on the sample table of scanning electron microscope (Hitachi, Tokyo, Japan). The surface morphology of polysaccharide was observed under the acceleration voltage of 30 kV.

### 4.7. UV and ATR-FTIR Spectra Analysis

The UV spectrum of polysaccharide samples was determined by a UV-1900i spectrophotometer (Shimadzu, Japan) with a scanning range of 200–400 nm. The attenuated total reflection (ATR) Fourier-transform infrared (FT-IR) spectrum of NPCP was analyzed by the Nicolet iS5 infrared spectrometer (Thermo Fisher Scientific, Waltham, MA, USA) in the wavenumber range of 4000–600 cm^−1^.

### 4.8. NMR Analysis

A total of 40 mg NPCP was dissolved in 0.5 mL D_2_O, and the ^1^H NMR, ^13^C NMR, ^1^H-^1^H correlation spectroscopy (COSY), total correlation spectroscopy (TOSCY) hetero-nuclear single quantum coherence (HSQC), and hetero-nuclear multiple bond correlation (HMBC) spectra were recorded by a 600 MHz NMR spectrometer (Bruker, Rheinstetten, Germany).

### 4.9. Immunomodulatory Activity of NPCP on Macrophages Polarization

#### 4.9.1. Cell Culture

The RAW264.7 cell line was obtained from the National Collection of Authenticated Cell Cultures (Shanghai, China). Cells were cultured in DMEM medium containing 10% FBS, 100 U/mL penicillin, and 100 mg/mL streptomycin at 37 °C in a 5% CO_2_ incubator.

#### 4.9.2. Cell Viability Assay

RAW264.7 cells were seeded in 96-well plates at 2 × 10^3^ cells/well and incubated at 37 °C in 5% CO_2_ incubator for 24 h. Then NPCP at final concentrations of 50, 100, and 200 μg/mL were added and incubated for another 48 h. The cell viability was detected at 450 nm using a multifunctional microplate reader (Allshen, Hangzhou, China) by the CCK8 assay.

#### 4.9.3. Phagocytic Activity Assay

RAW264.7 cells were seeded at 1 × 10^4^ cells/well in 96-well plates and incubated in culture medium at 37 °C for 24 h. After that, NPCP at final concentrations of 50, 100, and 200 μg/mL were added and incubated for another 24 h. Next, the cells were washed twice with PBS and then treated with 50 μL (0.15%) neutral red solution for 4 h. Finally, the absorbance was measured at 540 nm using a multifunctional microplate reader.

#### 4.9.4. Cytokine Assay

RAW264.7 cells were cultured in DMEM, LPS (10 μg/mL), IL-4, and IL-13 (20 ng/mL) for 24 h; then, NPCP at final concentrations of 50, 100, and 200 mg/mL was added to the LPS, IL-4, and IL-13 groups for another 24 h. The concentrations of IL-6, IL-10, and TNF-α in the supernatant were detected by ELISA kits according to the manufacturer’s instructions.

#### 4.9.5. Flow Cytometry

The cells of each group were collected and incubated with anti-CD86-FITC (eBioscience, San Diego, CA, USA) at 4 °C in the dark for 30 min. After washing with PBS, the supernatant was removed by centrifugation; then, anti-CD206 antibody was added and incubated at 4 °C in the dark for another 30 min. The same type of antibody was used as a negative control. Finally, the expression of CD86 and CD206 in each group of cells was measured by flow cytometry and analyzed with Flowjo software (version 10).

#### 4.9.6. Statistical Analysis

All experiments were repeated three times, and the results were expressed as mean ± standard deviation (SD). Statistical significance was analyzed by one-way analysis of variance (ANOVA) using SPSS software (version 25). *p*-Value < 0.05 was regarded as statistically significant.

## 5. Conclusions

In this study, a neutral polysaccharide NPCP was extracted and isolated from *Polygonatum cyrtonema*, and its structure was characterized using techniques such as HPGPC, GC-MS, FT-IR, SEM, and NMR. The structure of its sugar chain was speculated to be →1)-α-D-Glc-(4→1)-β-D-Gal-(3→. The effects of NPCP on macrophages polarization were investigated by CCK8 assay, neutral red assay, flow cytometry, and ELISA. Results showed that NPCP could promote macrophages proliferation and enhance macrophages phagocytosis activity. Moreover, NPCP exerted inhibitory effects on both M1 and M2a macrophages, leading to a reduction in the levels of IL-6 and TNF-α in these cells while simultaneously increasing the level of IL-10. These findings may reveal that NPCP is a potential immunomodulatory agent with promising anti-inflammation applications.

## Figures and Tables

**Table 1:** Methylation analysis results of NPCP.

Retention Time	Methylated Sugar	Type of Linkage	Mass Fragments (*m*/*z*)	Molar Ratios%
44.23	2,3,4,6-Me_4_-Galp	T-linked Galp	43, 87, 101, 129, 145, 161, 205	3.38
45.59	2,4,6-Me_3_-Galp	1,3-linked Galp	43, 87, 101, 117, 129, 145, 161, 205, 233	4.43
48.29	2,3,6-Me_3_-Glcp	1,4-linked Glcp	43, 87, 101, 117, 129, 161, 173, 233	47.35
49.02	2,3,4,6-Me_4_-Glcp	T-linked Glcp	43, 87, 101, 117, 129, 161, 173, 189, 203	8.01

**Table 2:** ^1^H and ^13^C NMR chemical shifts of NPCP.

Glycosyl Residues	Chemical Shift δ (ppm)						
	C1/H1	C2/H2	C3/H3	C4/H4	C5/H5	C6/H6	H6b
→4)-α-D-Glcp-(1→	5.32 92.11	3.47 70.69	3.66 72.67	3.86 73.72	3.76 73.26	3.84 60.42	3.69
→3)-β-D-Gal-(1→	4.56 103.77	3.61 71.96	4.07 76.69	3.69 72.81	3.77 72.51	3.66 62.48	3.58
α-D-Glcp-(1→	5.32 92.11	3.46 70.99	3.67 72.42	3.72 71.72	3.76 72.46	3.84 60.42	3.69
β-D-Gal-(1→	4.56 103.77	3.61 71.96	3.65 71.88	3.69 72.81	3.77 72.51	3.66 62.48	3.58

## Data Availability

The data presented in this study are available in article and Supplementary Material.

## Supplementary Materials

The following supporting information can be downloaded at: https://www.mdpi.com/article/10.3390/molecules29092076/s1, Figure S1: Chromatogram of Dextran standard. Figure S2: Methylation analysis chromatogram of NPCP. Figure S3: Mass fragments of methylation produce.
