# Supplementary material for: Structural Characterization of Polygonatum Cyrtonema Polysaccharide and Its Immunomodulatory Effects on Macrophages
Source: Molecules. 2024 Apr 30;29(9):2076. doi: 10.3390/molecules29092076 (PMC11085417; doi:10.3390/molecules29092076)
Supplement: Supplementary file 1 [file molecules-29-02076-s001.zip › molecules-2946044-supplementary.pdf]

Supplementary Material

Contents:

Figure S1. Chromatogram of Dextran standard.

Figure S2. Methylation analysis chromatogram of NPCP.

Figure S3. Mass fragments of methylation produce.

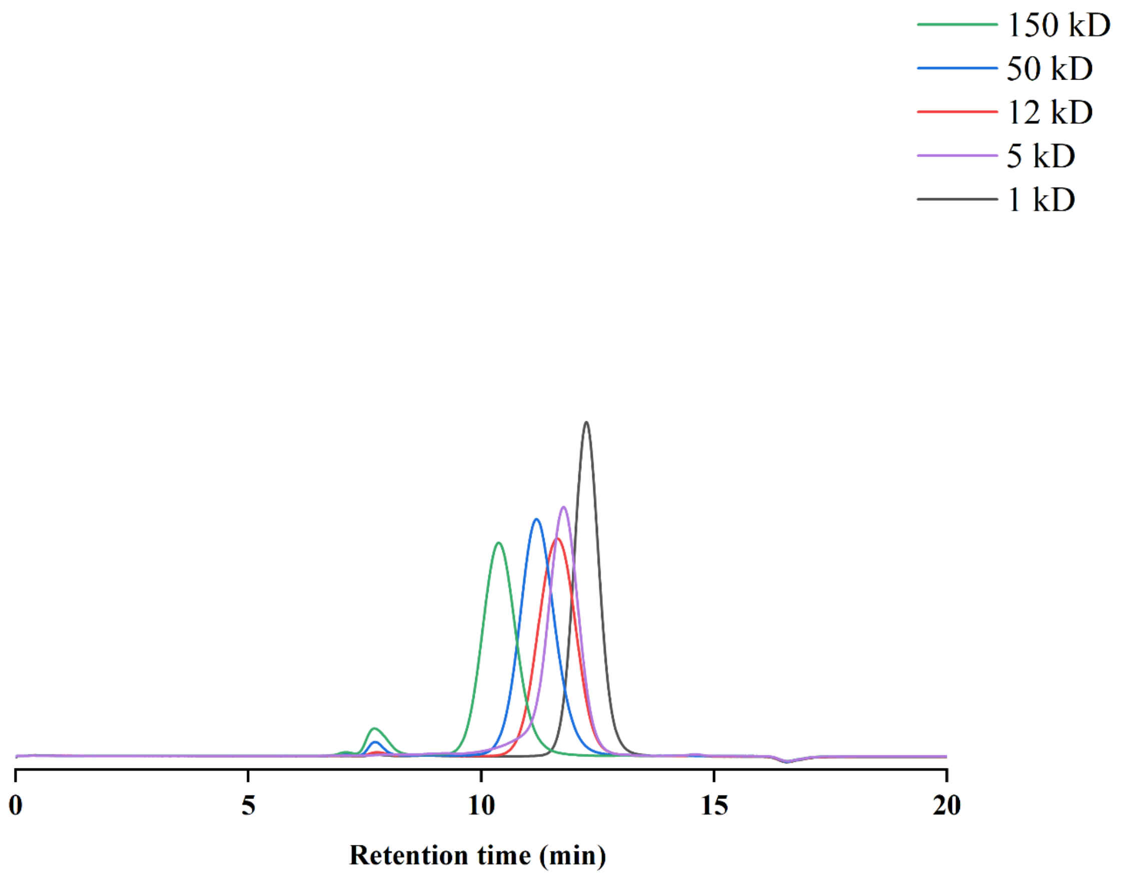

Figure S1. Chromatogram of Dextran standard.

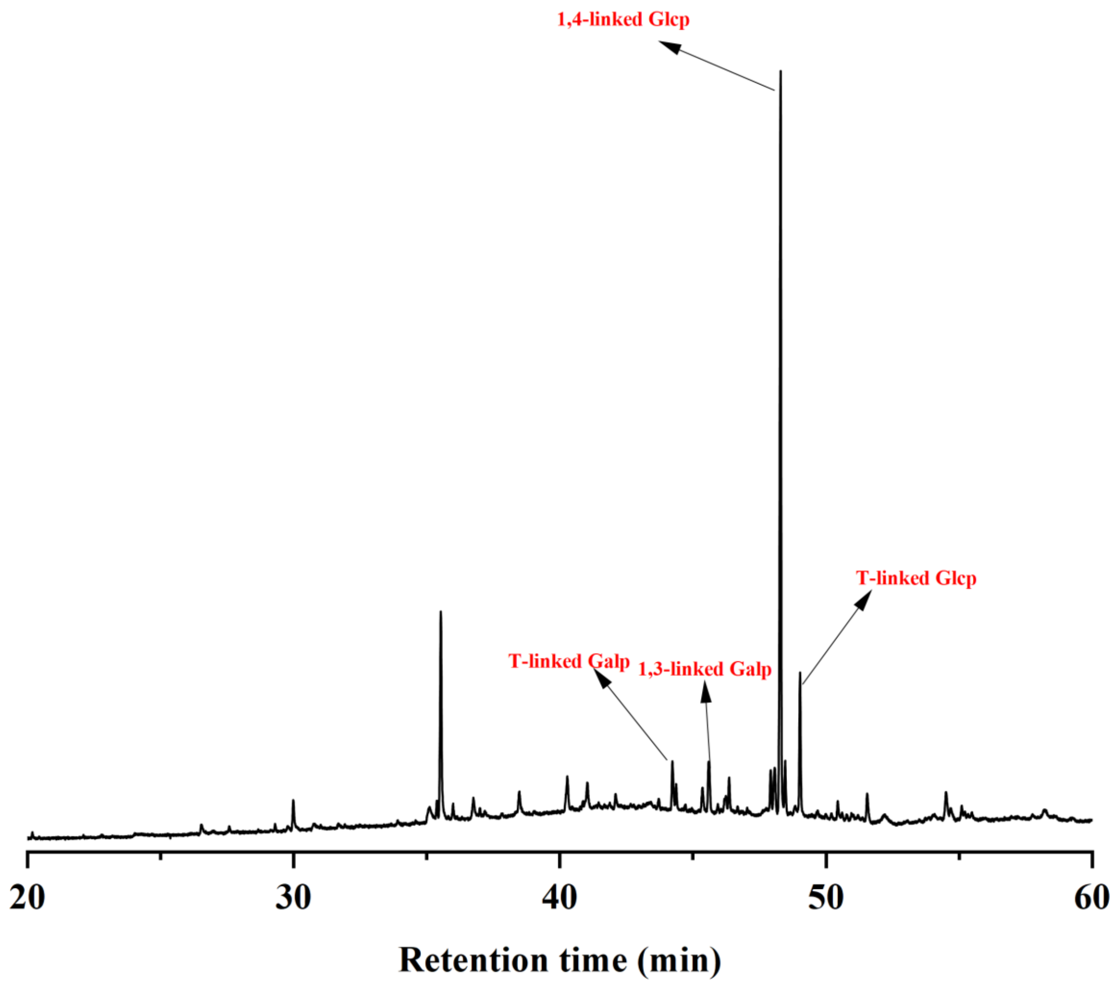

Figure S2. Methylation analysis chromatogram of NPCP.

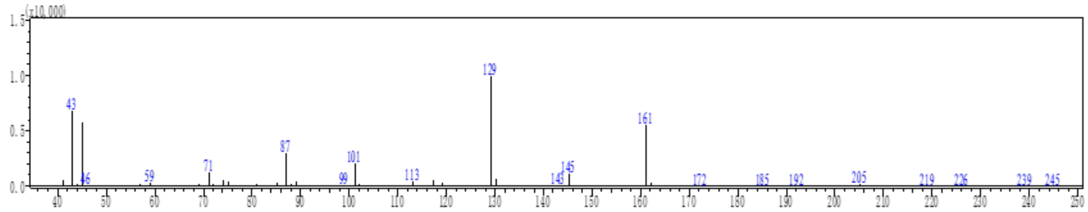

44.23 min

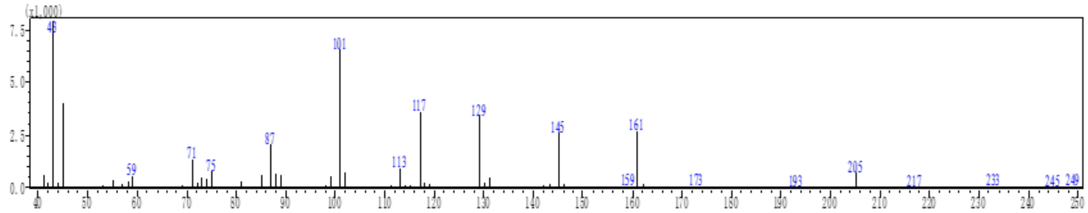

45.59 min

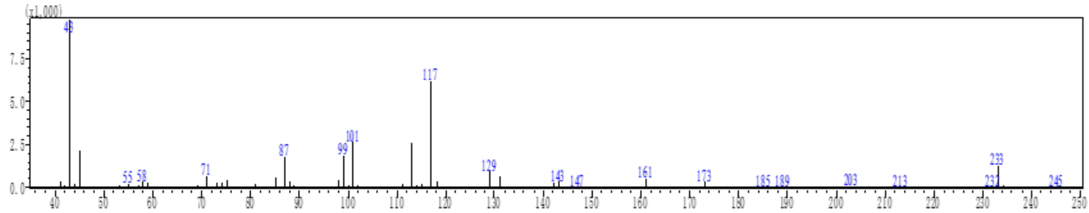

48.29 min

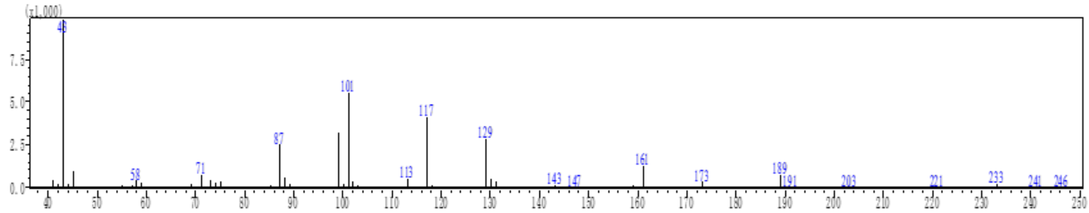

49.02 min

Figure S3. Mass fragments of methylation produce.
